# European recommendations on practices in pediatric neuroradiology: consensus document from the European Society of Neuroradiology (ESNR), European Society of Paediatric Radiology (ESPR) and European Union of Medical Specialists Division of Neuroradiology (UEMS)

**DOI:** 10.1007/s00247-022-05479-4

**Published:** 2022-09-05

**Authors:** Andrea Rossi, Maria Argyropoulou, Dora Zlatareva, Gregoire Boulouis, Francesca B. Pizzini, Luc van den Hauwe, Maria Raissaki, Jean-Pierre Pruvo, Karen Rosendahl, Chen Hoffmann, Pia C. Sundgren, ESNR Pediatric Neuroradiology Subspecialty Committee, Kshitij Mankad, Mariasavina Severino, ESPR Neuroradiology Taskforce, Catherine Adamsbaum, Irmhild Altmann-Schneider, Jean-François Chateil, Daniel J. Connolly, Volodia Dangouloff-Ros, Felice D’Arco, Veronica Donoghue, Nadine Girard, Sidney Krystal, Maarten Hans Lequin, Dhananjaya Narayana, Luciana Porto, Andrea Rossi, Elida Vasquez, Vasileios Xydis

**Affiliations:** 1 Neuroradiology Unit, IRCCS Istituto Giannina Gaslini, Via Gerolamo Gaslini 5, I-16147 Genoa, Italy; 2 Department of Health Sciences, University of Genoa, Genoa, Italy; 3 Department of Clinical Radiology and Imaging, Medical School, University of Ioannina, Ioannina, Greece; 4 Department of Diagnostic Imaging, Medical University Sofia, Sofia, Bulgaria; 5 Diagnostic and Interventional Neuroradiology Department, University Hospital of Tours, Tours, France; 6 Radiology Institute, Department of Diagnostic and Public Health, University of Verona, Verona, Italy; 7 Department of Radiology, Antwerp University Hospital, University of Antwerp, Antwerp, Belgium; 8 Deparment of Radiology, AZ Klina, Brasschaat, Belgium; 9 Department of Radiology, Medical School, University Hospital of Heraklion, University of Crete, Heraklion, Greece; 10 Diagnostic and Interventional Neuroradiology Department, University Hospital of Lille, Lille, France; 11 Research Group for Medical Imaging, University Hospital North Norway, UiT the Arctic University of Norway, Tromsø, Norway; 12 Department of Radiology, Tel Hashomer Hospital, Chaim Sheba Medical Center, Ramat-Gan, Israel; 13 Department of Radiology, Clinical Sciences Lund, Lund University, Lund, Sweden; 14 Department for Medical Imaging and Physiology, Skåne University Hospital, Lund, Sweden; 15 Lund University BioImaging Centre (LBIC), Lund University, Lund, Sweden; 16 Neuroradiology Unit, Great Ormond Street Hospital for Children, London, UK; 17 Pediatric Radiology Department, Bicêtre Hospital, AP-HP, Le Kremlin Bicêtre, France; 18 Paris-Saclay University, Le Kremlin Bicêtre, France; 19 Department of Radiology and Nuclear Medicine, Utrecht University Medical Center, Utrecht, The Netherlands; 20 Pediatric and Prenatal Imaging Department, Hôpital Pellegrin, CHU de Bordeaux, Bordeaux, France; 21 University of Bordeaux, UMR 5536, Bordeaux, France; 22 Department of Radiology, Sheffield Children’s NHS Foundation Trust, Sheffield, UK; 23 Department of Pediatric Radiology, Hôpital Necker Enfants Malades, Institut Imagine, Paris, France; 24 24 Radiology, National Maternity Hospital, Dublin, Ireland; 25 Department of Neuroradiology, APHM La Timone, Marseille, France; 26 Aix-Marseille University, Marseille, France; 27 Radiology Unit, Rothschild Foundation, Paris, France; 28 Abhayahasta Multispeciality Hospital, Bengaluru, Karnataka India; 29 Institute of Neuroradiology, University Hospital Frankfurt, Goethe University, Frankfurt am Main, Germany; 30 Department of Pediatric Radiology, Vall d’Hebron University Hospital, Barcelona, Spain

**Keywords:** Adolescent, Child, Education, Fetus, Guidelines, Imaging, Neuroradiology, Pediatric, Radiology, Recommendations, Standard of practice, Training

## Abstract

Pediatric neuroradiology is a subspecialty within radiology, with possible pathways to train within the discipline from neuroradiology or pediatric radiology. Formalized pediatric neuroradiology training programs are not available in most European countries. We aimed to construct a European consensus document providing recommendations for the safe practice of pediatric neuroradiology. We particularly emphasize imaging techniques that should be available, optimal site conditions and facilities, recommended team requirements and specific indications and protocol modifications for each imaging modality employed for pediatric neuroradiology studies. The present document serves as guidance to the optimal setup and organization for carrying out pediatric neuroradiology diagnostic and interventional procedures. Clinical activities should always be carried out in full agreement with national provisions and regulations. Continued education of all parties involved is a requisite for preserving pediatric neuroradiology practice at a high level.

## Introduction

This is a consensus document providing recommendations based on expert opinion and best available evidence, regarding the optimal conditions for the safe practice of pediatric neuroradiology.

## Article 1: definition

Pediatric neuroradiology is a subspecialty within the greater radiologic field, with possible career pathways to the discipline from radiology, neuroradiology and pediatric radiology. Pediatric neuroradiology involves imaging studies of the central and peripheral nervous system, the head, neck and spine, performed in fetuses, newborns, infants, children and adolescents. Most of these studies are diagnostic; however, some are interventional, including endovascular and percutaneous procedures performed to treat the patient’s condition with a minimally invasive approach that uses neuroradiologic imaging techniques as a guide.

## Article 2: general techniques and indications

A wide spectrum of imaging techniques is available for pediatric neuroradiology studies, including ultrasound (US), computed tomography (CT), magnetic resonance imaging (MRI), conventional radiography and digital subtraction angiography (DSA). The choice of the optimal imaging technique depends on several factors, such as age, degree of cooperation, clinical conditions and indications and available facilities.

Radiation protection comprises the principles of justification (i.e. a radiologic study should be performed only when the required relevant information cannot be obtained from existing charts or studies and should result in a net benefit to the patient) and optimization (the lowest possible radiation dose to obtain the relevant information should be administered) [1]. Since safety issues and radioprotection concerns are of the utmost importance in children, the choice of the imaging technique should, whenever feasible, favor modalities that do not use ionizing radiation (such as US and MRI) over those that do (such as radiographs and CT). Whenever ionizing radiation is used, the ALARA principle (“as low as reasonably achievable”) should always be applied [2, 3].

In fetuses, newborns and infants, and until the fontanelles are closed, US of the brain coupled with color Doppler is the first-line imaging modality (except for trauma), followed by MRI when necessary [4, 5]. For trauma, CT is the method of choice. For the neonatal spinal canal, US is also the first-line examination offering not only morphological information but also real-time information on the systole-diastolic motion of the spinal cord and the nerve roots [6]. An MRI of the spine follows, if necessary.

In children over 1 year of age, MRI is the examination of choice for imaging the brain and intraspinal structures, while radiography is the initial examination for some spine-related problems, such as scoliosis, kyphosis and persistent back pain, or to assess spinal stability [7–9].

CT is performed in specific indications, mainly involving the study of bony structures such as the temporal bone as well as in head trauma. For the study of superficial structures in the head and neck, including the thyroid, an US coupled with color Doppler is the preferred initial examination, and if necessary further characterization and mapping is performed with MRI [10, 11].

Conventional radiography of the skull is still used as an initial screening test for indications like “lumps and bumps” [12], for non-syndromic craniosynostosis [13] and as part of a skeletal survey when examining for skeletal dysplasias.

Digital subtraction angiography (DSA) is commonly used in the context of endovascular interventional procedures, for the presurgical planning of interventions involving cervical or intracranial vessels and for a few residual diagnostic indications when a diagnostic angiogram obtained noninvasively with MR angiography or CT angiography has an inferior diagnostic accuracy [14–16].

## Article 3: site conditions

The practice of pediatric neuroradiology should preferably take place in health care institutions that routinely provide services and treatments during pregnancy and childhood (as defined in Article 1). These institutions may be tertiary care pediatric hospitals or general hospitals in which pediatric health care services are available.

Facilities that should optimally be available on site include:

- Emergency department.
- Inpatient hospital wards and beds.
- In a tertiary care pediatric hospital, a pediatric imaging department comprising units or divisions of pediatric radiology and pediatric neuroradiology employing trained specialists and a team of trained interventional radiologists/neuroradiologists performing endovascular and percutaneous procedures.
- State-of-the-art equipment including radiography, US, CT and MRI facilities as well as an angiographic suite.
- A department of pediatrics with neonatal and pediatric intensive care units and ward.
- Access to genetics and metabolic medicine.
- Research facilities (in academic centers).

Work schedules can be organized into daily routines and either on-site or on-call night/holiday shifts depending on the workload. In any case, access to pediatric neuroimaging studies should be available 24 h a day, every day (24/7). The institution should provide a minimal individual workload to be recognized and certified in the practice of pediatric neuroradiology according to the national and international recommendations for each technique. Collaboration of different institutions on a local/metropolitan basis may be employed to guarantee 24/7 coverage, especially during night shifts, when single institutions are unable to provide such coverage individually. Teleradiology services may also be employed to overcome local center shortages, provided that such services are able to ensure required quality standards. Safe practices specific for each technique should be guaranteed and always reinforced, with specific focus on radioprotection and continuing medical education, as well as on meeting and maintaining national and international regulations.

## Article 4: team requirements

Requirements for team composition vary depending on the imaging modality. While all radiologic specialists are, by definition, entitled to read and report pediatric neuroradiology studies, the scientific societies and bodies that endorse the present document (European Society of Neuroradiology [ESNR], European Society of Paediatric Radiology [ESPR] and European Union of Medical Specialists [UEMS] Division of Neuroradiology) advocate that pediatric neuroimaging studies are carried out by specialists in neuroradiology or pediatric radiology who preferably have completed formal training in the field of pediatric neuroradiology. However, we acknowledge that, at the time of the extension of this document, formalized pediatric neuroradiology training programs are not available in the majority of European countries, and even worldwide; consequently, while we advocate that such programs should rapidly become widespread, we also recommend that, in the absence of a specific training program, pediatric neuroradiology studies be performed and reported by radiologists or neuroradiologists who have achieved an established experience in their fields.

To support pediatric health care, European scientific societies are making large efforts to achieve training and certification of pediatric neuroradiology specialists; ESNR and ESPR are jointly organizing the European Course on Pediatric Neuroradiology, structured as a biennial two-module course comprising in-person lectures and interactive case-based workshops; furthermore, the European Diploma in Pediatric Neuroradiology (EDiPNR), a certificate of excellence administered by the European Board of Neuroradiology (EBNR) and endorsed by ESNR, ESPR and UEMS Division of Neuroradiology, certifies a candidate’s competence in pediatric neuroradiology in a standardized way across Europe [17]. Furthermore, both the European Diploma in Pediatric Radiology (EDiPR, ESPR) and the European Diploma in Neuroradiology (EDiNR, ESNR) include significant pediatric neuroradiology teaching, paving the way for further super-specialist training fully available with the EDiPNR.

The specificities of pediatric interventional neuroradiology (INR) make it advisable that operators performing DSA as well as interventional endovascular procedures in children be fully trained on adult procedures and maintain their endovascular skills through high-volume children-adult INR platforms. Regarding technical and personnel requirements for pediatric DSA, which are usually an integral part of planning, performance and monitoring of endovascular treatment, we refer to the UEMS documents on the training and practice of interventional neuroradiology in Europe [18, 19].

Pediatric neuroradiologists should be part of a team that also includes pediatric nurses and radiographers. In some centers, US studies may be carried out by trained pediatric radiographers/sonographers under supervision of a pediatric radiologist or neuroradiologist. Non-radiologic point-of-care ultrasound (POCUS) may represent a viable solution where radiologic resources are limited, but it requires good training and an accreditation and governance structure, so as to avoid erroneous diagnoses, poor outcomes and litigation [20].

Pediatric neuroradiologists should be encouraged to organize and lead multidisciplinary team meetings to discuss imaging findings with pediatricians, pediatric neurologists, neurosurgeons, oncologists, geneticists and other specialists as required [21].

## Article 5: uncooperative patients

The possibility of routinely accommodating sedation and/or general anesthesia should be guaranteed for each technique except radiography and US. Assistance from pediatric anesthesiologists is required for sedation procedures in several European countries, but protocols for sedation vary across institutions.

In general, the need for sedation/anesthesia is especially relevant for uncooperative children (usually under the age of 5 years) undergoing MRI; specific preparation (mock MRI, video tutorials, visits to the CT or MR suite the day before the appointment, etc.) is advised to reduce the need for sedation in younger patients and to counteract anxiety in older, potentially cooperative children. Distraction strategies, such as movies or virtual reality devices specifically conceived for children, can effectively decrease anxiety and significantly improve the patient’s experience during the MRI examination.

For neonatal MRI studies, a feed-and-wrap/swaddle technique with the scan being coordinated with the child’s biorhythms can be successfully applied in most instances, thereby obviating the need for sedation [22]. This technique can also be successfully employed to manage a large proportion of uncooperative patients undergoing CT, coupled with the extremely rapid acquisition times permitted by up-to-date CT scanners [23].

Respirators and other anesthesia equipment should always be available for all patient sizes and should be operated by anesthesiologists specifically trained to manage children. For sedations in the MRI suite, all employed instrumentation and equipment must be MR compatible.

The pediatric radiology/neuroradiology department should be equipped with adequate waiting areas and recovery rooms where patients can be prepared for and observed after execution of the procedures. Attention should be given to creating a child-friendly and pleasant environment, with special decorations to make children feel more comfortable and decrease their anxiety. Specially trained radiographers and nurses should be available.

All angiographic procedures, either diagnostic or interventional, must be performed under sedation in pediatric patients.

## Article 6: recommendations on postnatal imaging modalities and protocols

### Ultrasound

Ultrasound (US) is the first-choice imaging modality to evaluate the infantile brain provided that a state-of-the-art technique is applied [24, 25]. The following is recommended:

- A gray-scale color Doppler US machine equipped with sectorial and linear transducers: More precisely, a small sector transducer (5 to 8 MHz) to evaluate the whole brain and a linear-array transducer (5 to 12 MHz) to study the morphology and echo structure of the brain and the peri-cerebral spaces, and to assess patency and flow characteristics of vascular structures.
- Use of acoustic windows: The anterior fontanelle is the main acoustic window; however, the use of accessory acoustic windows such as the posterior fontanelle, the mastoid fontanelle and (in rare situations) the foramen magnum can be useful in selected indications to provide high-quality images of regions that are distant to the anterior fontanelle (e.g., the posterior cranial fossa) [26, 27].
- A standard protocol should be performed:
  - Using the sectorial transducer, coronal scans (ideally, five to seven) and sagittal scans (ideally, five) should be performed through the anterior fontanelle to evaluate the whole brain. Using the linear transducer, at least one midline sagittal and one coronal scan should be performed to appreciate the gray to white matter differentiation and for a detailed evaluation of the corpus callosum, basal ganglia, brainstem, vermis and extra-axial cerebrospinal spaces. A detailed evaluation of the occipital lobes and cerebellum should be performed through the posterior and the mastoid fontanelle (at least one coronal and one axial scan are recommended).
  - Pulsed color Doppler should be systematically performed especially in preterm babies [28]. Brain arteries are low-resistance vessels presenting a positive systolic and diastolic flow. The resistive index (RI) should be measured, and in case of hydrocephalus the delta RI (i.e., calculated from RI with and without compression to the anterior fontanelle) should be ascertained before any therapeutic drainage [28, 29]. Venous sinuses and the thalamostriate and internal cerebral veins can be easily evaluated with US. Lack of flow in a terminal vein in a preterm baby with intraventricular hemorrhage heralds the development of a homolateral periventricular venous infarct. The application of color Doppler at the foramina of Monro and the aqueduct of Sylvius by depicting alternating blue and red color echoes suggests particles in the cerebral spinal fluid (CSF), such as blood cells compatible with subarachnoid hemorrhage [28].
- US is also the first-choice imaging modality to evaluate the infantile spinal canal [30]. It offers morphological and motion information on the spinal cord and the nerve roots. In neonatal and early infantile life, the spinal arches are mainly cartilaginous and thus offer an adequate acoustic window. Sagittal and axial scans should be performed depicting the normal hypoechoic spinal cord, the hyperechoic central canal and the hyperechoic nerve roots.

Indications for head and neck US include:

- Congenital malformations (e.g., corpus callosum agenesis, holoprosencephaly, etc.) and infections (e.g., TORCH).
- Acquired infections (e.g., meningoencephalitis, abscess).
- Encephalopathy of prematurity: periventricular leukomalacia, intraventricular hemorrhage with its complications (such as venous infarct and hydrocephalus).
- Encephalopathy of the full-term baby due to hypoxia-ischemia and stroke (arterial or venous).
- Superficial masses of the head and neck [31].
- Congenital hypothyroidism [11].
- Increasing head circumference for the exclusion of hydrocephalus, extra-axial fluid/collections, and high flow arteriovenous shunts such as vein of Galen aneurysmal malformation, dural sinus malformation and pial arteriovenous fistulae.

Indications for spinal canal US include:

- Congenital anomalies (such as the various forms of spinal dysraphism).
- Caudal regression syndrome (e.g., anal atresia or stenosis; sacral agenesis).
- Acquired abnormalities (spinal cord injury, meningitis, hemorrhage).
- Neuroblastoma extension into the spinal canal.
- Guidance for lumbar puncture.

### Computed tomography

In the pediatric age group, CT should be used with caution and indications should always be carefully weighed in view of radioprotection issues [32]. Dual-energy CT and spectral CT or, if not available, multi-detector row CT scanners adopting dose-reduction techniques should be employed [33]. Special consideration should be devoted to reducing the dose for indications in which the primary anatomy of interest does not require the same level of image quality as routine brain CT scans, such as craniosynostosis and hydrocephalus follow-up [34, 35].

Indications for CT include:

- Craniofacial and spinal trauma.
- Syndromic craniosynostosis and craniofacial malformations.
- Search for intracranial calcifications.
- Temporal bone and skull base abnormalities.
- Presurgical assessment for nasal/paranasal sinus surgery.
- CT angiography: hemorrhagic stroke, vascular malformations.
- Vertebral bone abnormalities, especially for preoperative assessment of scoliosis.
- Unavailability of MRI (i.e. technical failure).

As a rule and with the exception of the above indications, CT scanning should not be routinely used as a first-line imaging modality for brain and spine evaluation in children. However, CT can help clarify findings in case of equivocal MRI results.

Acquisition of the volume of interest occurs on the axial plane and is followed by orthogonal coronal, sagittal or three-dimensional reformats, as required. It is fundamental to always analyze not just the soft-tissue window but also the bone window.

Iodinated contrast material for intravenous injections can be used to clarify or further investigate findings whenever necessary. The contrast media concentration should be 300 mg/ml and a 2 ml/kg patient weight dose should be used. The contrast material is injected intravenously, either manually or via a power injector. The latter should be used for CT angiography procedures. Contrast-enhanced acquisitions should be preceded by an unenhanced study, otherwise findings may not be correctly interpreted, a typical instance being the differentiation of a spontaneous hyperdensity (i.e. hemorrhage) from pathological enhancement (i.e. blood-brain barrier disruption).

### Magnetic resonance imaging

Magnetic resonance imaging is the method of choice for a variety of indications from fetal life to adolescence. In newborns, both premature and term, an additional MRI may clarify equivocal US/Doppler findings. In infants, after closure of the fontanelles, MRI represents the main modality for imaging of the brain and spinal canal.

Indications for MRI include but are not limited to:

- Fetal MRI (malformations, acquired fetal damage).
- Emergent neuroimaging in patients presenting with acute neurological illness.
- Trauma (as a complementary modality), including suspected abusive head trauma and post-delivery brachial plexus palsy.
- Epilepsy.
- Stroke and vascular abnormalities.
- Brain and spine malformations.
- Pathologies of the peripheral nerves.
- Genetic disorders presenting with developmental delay or autistic spectrum disorder.
- Neurocutaneous and other genetic syndromes.
- Infectious, inflammatory and demyelinating diseases.
- Metabolic and neurodegenerative diseases.
- Brain and spinal tumors.
- Hypothalamic-pituitary, orbital, and head and neck disorders including inner ear pathologies.

Health care centers providing pediatric neuroradiology services should be equipped with state-of-the art MR scanners. Although many initial pediatric neuroradiology exams are performed in nonspecialized centers that may not be perfectly equipped, magnets of lower field strength than 1.5 Tesla (T) should not be used for pediatric neuroimaging. Ideally, 3-T magnets should be preferred over 1.5-T scanners for brain MRI, owing to their superiority in terms of spatial and contrast resolution [36]. This is especially valid in special conditions, such as focal epilepsy in which causal lesions may not be easily detected at lower field strength. For advanced MRI studies, such as those involving blood oxygenation level dependent (BOLD) functional MRI (fMRI), multidirectional diffusion-weighted imaging (DWI) (including but not limited to diffusion tensor imaging), proton MR spectroscopy and perfusion-weighted imaging [37], 3-T magnets are also preferred.

In general, the longer duration of MRI studies over CT is often perceived as a limitation, especially in unstable or uncooperative patients in emergency situations. However, fast imaging protocols, which obtain MR sequences in a matter of a few seconds (such as the echoplanar single-shot T2-weighted sequences and others), have expanded the indications for MRI in the uncooperative child and provide a valid alternative to CT, especially when considering radioprotection issues and the risks related to multiple exposures (a typical example being the follow-up of patients with hydrocephalus and ventricular shunts) [37–40]. Newer sequences, such as the black-bone technique, also offer alternatives to CT in neurotrauma and hydrocephalus [38–40].

Protocols for pediatric neuro-MRI should be tailored according to clinical indication and patient age. Myelination is a maturational process of the white matter that begins in utero and proceeds until well into the third year of life in an orderly and fully predictable manner. The unmyelinated white matter is rich in free water, whereas myelinated white matter is mostly comprised of multilayered proteolipid membranes with little intervening free water. In the fully myelinated brain, it is recommended that MRI protocols include T1-, T2- and fluid-attenuated inversion recovery (FLAIR) sequences obtained in the three planes of space, in addition to axial DWI and susceptibility-weighted images (SWI). An ideal baseline protocol will typically include 3-D FLAIR and 3-D T1 sequences with triplanar reformats, as well as 2-D T2-weighted images obtained at least in the axial and coronal planes and axial DWI and susceptibility weighted imaging (SWI). Other sequences depending on the clinical indication may include, but are not limited to, high-resolution T2-weighted sequences, black-blood T1-weighted sequences, DTI, MR angiography and MR venography. Fat suppression techniques can be used for several indications; however, a non-fat-saturated sequence should always be included for comparison.

Neonatal MRI studies should include modified T1- and T2-weighted images in which the repetition time (TR) and echo time (TE) are adequately increased to reflect the water-rich environment of the unmyelinated brain [41, 42]. On the other hand, FLAIR images obtained in neonates and infants younger than 1 year notably suffer from lack of contrast between gray and white matter, exhibit a triphasic sequence of relative white matter signal change during myelination [43] and are thus seldom useful, except for the study of pathologies involving the subarachnoid spaces and meninges [44].

The use of gadolinium-based contrast agents (GBCA) represents an important component of MRI studies performed in children for indications in which blood-brain barrier damage is present or suspected, such as brain and spine tumors, infectious and inflammatory disorders, neurocutaneous disorders (when there is a query for associated tumor) and other conditions [44]. The intravenous injection of GBCA should always be carefully considered in the face of safety concerns, including the issues of gadolinium retention, nephrogenic systemic fibrosis (NSF) associated with GBCA and renal immaturity (a physiological phenomenon in neonates and infants) [45]. At present, regulations regarding the intravenous administration of GBCA differ in various countries. In Europe, the European Medicines Agency has proscribed the use of linear agents for neuroimaging procedures, so that only macrocyclic agents having highest chelate stability may be used [45]. Notably, there are restrictions in the type of macrocyclic GBCA that can be used in neonates and infants up to 6 months old in several European countries. Pediatric neuroradiologists should be aware of these limitations and consider them in the choice of the GBCA to be used. In general, however, there has been an increased caution and, consequently, a greater restraint in the liberal use of GBCA for pediatric indications. Several conditions, such as neurofibromatosis, tuberous sclerosis or certain types of tumors such as craniopharyngiomas or other low-grade tumors, are now frequently followed up without the routine use of contrast agents.

### Radiography

Radiography in children should be performed by radiographers with knowledge of age-specific radiation issues, positioning, distracting and restraining techniques as well as age-specific kV/mAs settings [46]. Indications have progressively diminished in recent years and are presently limited to spine radiographs for scoliosis and back pain, dynamic studies for suspected craniocervical instability and the diagnosis of osteoporosis. In some conditions, skull “lumps and bumps” and suspected non-syndromic craniosynostosis may be imaged with skull radiographs [47]. Craniospinal radiography is also part of the skeletal survey when imaging for suspected child abuse or skeletal dysplasias. For polytrauma, however, CT is the imaging method of choice. Knowledge of normal patterns of bone maturation, the presence and temporal evolution of sutures and normal variants is essential to correctly interpret craniospinal radiographs.

### Digital subtraction angiography

As a rule, the pediatric use of DSA should be reserved for interventional procedures or for the presurgical planning of interventions involving cervical and/or intracranial vessels, whenever diagnostic angiograms obtained either with CT angiography or MR angiography have inferior diagnostic capabilities [48].

Indications for DSA may include:

- Etiological workup of pediatric intracerebral hemorrhage, in case noninvasive imaging, including CT angiography and MR angiography, is inconclusive [14].
- Endovascular reperfusion techniques for arterial ischemic stroke (e.g., mechanical thrombectomy) [49].
- Neonatal vascular malformations (e.g., vein of Galen aneurysmal malformation, dural sinus malformation, etc.).
- Pediatric arteriovenous malformations and aneurysm.
- Preoperative brain tumor embolization (i.e. choroid plexus papilloma).
- Preoperative assessment of chronic intracranial arteriopathies (e.g., moyamoya disease) for vessel and anastomoses mapping.
- Angiographic test occlusion of a cervical or intracranial artery for surgical planning when a vessel sacrifice is anticipated.
- Head and neck vessel hemostatic embolization.
- Percutaneous sclerotherapy or (presurgical) embolization of craniofacial vascular malformations.

It is anticipated that indications for endovascular treatment of some conditions, prominently including the management of arterial ischemic stroke, will likely increase, thereby necessitating appropriate resource allocations and specifically trained management teams, at least in tertiary care referral centers.

## Article 7: recommendations for fetal imaging

Ultrasound is the screening modality of choice for fetal imaging. The method may, however, be limited by fetal positioning, maternal body habitus, oligohydramnios and limited field of view. In these cases, an additional MRI should be considered [50, 51]. Fetal postmortem MRI can also be performed as an adjuvant tool to intrauterine fetal MRI and conventional brain autopsy after termination of pregnancy and in stillborn fetuses [52, 53].

Indications for fetal neuroimaging include:

- Ventriculomegaly.
- Brain malformations (i.e. corpus callosum agenesis, holoprosencephaly, malformations of cortical development, midbrain-hindbrain malformations, cephaloceles, etc.).
- Intracranial solid or cystic masses.
- Family history of tuberous sclerosis.
- Vascular abnormalities (including vascular malformations, infarctions, hemorrhage, etc.).
- Monochorionic twin pregnancy complications.
- Vascular or lymphatic anomalies of the head and neck.
- Teratomas.
- Facial clefts.
- Spine and spinal cord malformations.
- Sacrococcygeal teratoma.

Prenatal US should be performed by experienced, dedicated specialists who have been properly trained in the specific field. Both transabdominal and transvaginal probes should be available.

Fetal MRI can be contemplated at any pregnancy stage to confirm anomalies detected by US, according to the American College of Radiology [54] and American College of Obstetricians and Gynecologists [55] consensus documents. Typically, however, fetal MRI is performed after the 19th gestational week and up to term, depending on the pathology suspected or national legislation that regulates elective termination of pregnancy; for some abnormalities, such as corpus callosum agenesis or ventriculomegaly, early detection is possible. For other pathologies, though, such as cortical malformations or the assessment of acquired lesions, third-trimester scanning (ideally between the 28th and 32nd weeks of gestation) may be required [56].

Scanners should be 1.5 or 3 T, with adequate specific absorption rate thresholding; notably, safety profiles for prenatal exposures to 3-T MRI have been shown to be excellent regardless of gestational age, both in terms of subsequent fetal growth and neonatal hearing, while spatial resolution and visualization of anatomical structures are improved and acquisition times are reduced at 3 T compared to 1.5 T [57].

Pregnant patients are preferably scanned in the lateral decubitus to avoid compression of the inferior vena cava. Standard fetal sequences include single-shot fast spin echo T2-weighted sequences and steady-state sequences (balanced turbo field-echo, true FISP, etc.), which should be acquired in the three planes of space, repeating the acquisition wherever necessary to compensate for fetal motion. Fetal brain studies should also include at least an axial DWI and an axial T1-weighted sequence (whose technical features depend on the individual scanner). Other fetal sequences, such as FLAIR, T2*-weighted images and others, can be employed in selected cases.

GBCAs must not be administered to pregnant patients unless considered essential [58].

CT scanning of the fetus is expressly forbidden for neurological indications. Low-dose (i.e. < 5 mSv) fetal CT can, however, be considered a complementary technique in the third trimester for the study of skeletal dysplasia or craniosynostosis [59].

## Conclusion

The present document serves as guidance for the optimal setup and organization of pediatric neuroradiological procedures in the diagnostic and interventional fields. The clinical activity should always be carried out in full agreement with national provisions and regulations. Special care for continued education of all parties involved is a requisite to maintain pediatric neuroradiology practice at a high level.
